# Results of Multigene Panel Testing, Including *PKD1*, in >1,200 Patients With Cystic Kidney Disease: A Retrospective Analysis

**DOI:** 10.1016/j.xkme.2025.101186

**Published:** 2025-11-13

**Authors:** Erin E. Tapper, Johanna M. Huusko, Alicia M. Scocchia, Kimberly Gall, Mary-Beth Roberts, Manuel Bernal-Quirós, Satu Valo, Inka Saarinen, Matias Rantanen, Tuuli Pietila, Massimiliano Gentile, Lotta Koskinen, Meenakshi Mahey Kumar, Samuel Myllykangas, Juha Koskenvuo

**Affiliations:** a Genetic Counseling Graduate Program, Augustana University, Sioux Falls, SD; b Blueprint Genetics, a Quest Diagnostics Company, Espoo, Finland; c Center for Personalized Genetic Healthcare, Medical Specialties Institute, Cleveland Clinic, Cleveland, OH

**Keywords:** AD-PKD1, chronic kidney disease, copy number variant, cystic kidney disease, genetic testing, massively parallel sequencing, molecular analysis, multigene panel testing, nephrology, nephrogenetics, next-generation sequencing, PKD1, polycystic kidney disease, pseudogene, variant of uncertain significance

## Abstract

**Rationale & Objective:**

Mounting evidence supports that identifying the specific molecular etiology for individuals with cystic kidney disease (CyKD) is important for prognostication, surveillance, identifying related living donors, and defining familial risk, even in cases in which a clinical diagnosis appears straightforward. In this study, we aimed to investigate the yield of genetic findings and the unique variant characteristics using multigene panel testing (MGPT) in a referral laboratory setting for an unselected population of patients with an indication of CyKD.

**Study Design:**

Cross-sectional study.

**Setting & Participants:**

A retrospective analysis of 1,235 genetic testing reports from patients with suspected CyKD who pursued MGPT was performed.

**Findings:**

A positive result in a gene associated with CyKD was identified in 49.4% (610/1235) of patient reports, identifying 468 unique variants classified as pathogenic or likely pathogenic in 20 unique genes. Variants in *PKD1*, a gene complicated by homology to 6 different pseudogenes, contributed to 65.6% (400/610) of positive results. Copy number variants (CNVs) were identified in 9.5% (58/610) of positive results, with 30.4% (17/56) of deletions consisting of 4 exons or less. Variants of uncertain significance that were suspicious for being pathogenic (susVUS) were identified in 57.0% (94/165) of patients with inconclusive results.

**Limitations:**

Genetic analysis was targeted to the genes included on the panel at the time of testing. As new evidence emerges supporting additional gene-disease associations, there is potential for additional positive results.

**Conclusions:**

Thoughtful selection of carefully curated MGPT optimized to detect technically challenging variants can identify the molecular etiology in individuals presenting with CyKD. Further investigation of susVUS through segregation analysis in families may contribute to additional positive results.

## Plain-Language Summary

Data informing the yield of multigene panel testing (MGPT) for individuals with cystic kidney disease (CyKD) is increasing. In this study, we retrospectively reviewed MGPT results from 1,235 individuals with suspected CyKD. A positive result in one of 20 CyKD-associated genes, including *PKD1*, was identified in 49.4% (610/1235) of patient reports. Copy number variants (CNVs) accounted for 9.5% (58/610) of positive results, with 30.4% (17/56) of deletions consisting of 4 exons or less. Suspicious variants of uncertain significance were identified in 57.0% (94/165) of patients with inconclusive results. MGPT can identify the molecular etiology of CyKD and, prior to ordering, should be carefully evaluated for relevant gene content, capabilities for technically challenging genes like *PKD1,* sensitivity to detect CNVs, and reporting policy for variants of uncertain significance.

Cystic kidney diseases (CyKD) are a heterogeneous group of inherited and sporadic conditions characterized by the formation of fluid-filled cysts in the kidney that lead to progressive kidney dysfunction. These conditions are collectively one of the most common causes of advanced kidney disease and kidney failure, and up to 25% of patients with advanced kidney disease have a genetic cause for their condition.^1^^,^^2^ Many hereditary CyKD conditions have associated extrarenal features that may be subclinical, for which early identification and surveillance can decrease morbidity and mortality.^3^

Genetic testing has historically not been part of the diagnostic work-up of CyKD, particularly autosomal dominant polycystic kidney disease (ADPKD) attributed to variants in the *PKD1* and *PKD2* genes because the diagnosis was traditionally made based on clinical presentation, imaging studies, and a positive family history.^4^ To date, many nephrologists have not incorporated genetic testing as a routine part of their clinical practice.^5^ Potential barriers to the adoption of genetic testing in the diagnostic workflow include cost of testing, a lack of perceived clinical benefit to the patient, uncertainty about which genetic test to choose and which patients may benefit from testing, unfamiliarity with interpretation and complexity of results, and a lack of expertise and training in incorporating genetic analysis into patient care.^6^, ^7^, ^8^

Identifying the molecular etiology of a patient’s CyKD has clinical utility, including implications for prognostication and surveillance recommendations, even in individuals for whom a clinical diagnosis is straightforward.^9^ There is also benefit for individuals without a positive family history or with an atypical presentation for whom making a clinical diagnosis is challenging, individuals for whom there is desire for prenatal or preimplantation genetic testing, or individuals for whom there is the need to confidently identify at-risk family members and related but unaffected donor candidates. Additionally, referrals for assessment and evaluation of extrarenal features may allow for proactive management.^10^, ^11^, ^12^, ^13^, ^14^ In recognition of the potential utility gained with identifying molecular etiology, the recent Kidney Disease: Improving Global Outcomes (KDIGO) guideline suggests incorporating the causal gene into the disease nomenclature for individuals diagnosed with ADPKD and have a pathogenic variant in *PKD1*, *PKD2*, or a gene for which pathogenicity is well supported (eg, ADPKD-PKD1).^15^

The accessibility and efficacy of genetic testing as a diagnostic tool for patients undergoing evaluation for CyKD has evolved over time. Historically, genetic testing for CyKD was limited to sequential testing of a small number of genes such as *PKD1*, *PKD2*, and *PKHD1*. Analysis of *PKD1* was challenging and laborious given the presence of 6 pseudogenes sharing significant sequence homology with the functional gene. In addition, detection of sequence variants and copy number variants (CNVs) was often done separately using different technologies, which was both time-consuming and costly. Although the number of genes associated with CyKD has grown to >100, the sequential, gene-by-gene, approach to genetic testing has become prohibitive.^16^, ^17^, ^18^ The availability of massively parallel sequencing, a testing methodology with the ability to sequence multiple genes in a single test and to assess for both sequence variants and CNVs simultaneously, has revolutionized genetic medicine and increased the utility of genetic testing for the diagnosis and management of CyKD.^19^^,^^20^ Multigene panel testing (MGPT) for CyKD is readily accessible at many accredited clinical genetic testing laboratories; however, important differences can exist between laboratories including panel gene content, technical capabilities and limitations, and laboratory reporting policies. It is critical that ordering providers recognize this variability and understand the implications of these differences when selecting testing for their patient and to accurately interpret a negative result.

Multiple publications have described the genetic findings in patients with chronic kidney disease.^19^^,^^21^^,^^22^ However, the genetic findings in a large cohort of individuals tested for the indication of CyKD, but unselected for age and inheritance, are limited.^19^^,^^20^^,^^23^, ^24^, ^25^, ^26^ In this study, we aimed to investigate the yield of genetic findings and unique variant characteristics using MGPT in a referral laboratory setting for an unselected population of patients with suspected CyKD. We present the rate of positive results and the genetic findings of MGPT in a population of individuals with CyKD to further the understanding of the genetic results that the patient’s care team can expect to see in their practice.

## Materials and Methods

Clinical reports for consecutive patients suspected to have CyKD and who underwent MGPT at Blueprint Genetics, a Clinical Laboratory Improvement Amendments (CLIA)-certified laboratory, were examined. Clinicians reported patient phenotypic information via the laboratory’s test requisition form. For the purposes of this study, an adult patient was defined as an individual 19+ years of age at the time of testing and a child patient was defined as an individual from birth to 18 years of age at the time of testing. Fetal samples are those from which DNA was extracted from tissue from amniocytes, chorionic villi, or fetal tissue obtained at fetopsy. Informed parental or personal consent for diagnostic genetic testing was obtained for all patients included in this study. This retrospective cross-sectional study of patient data was given an exempt status by the WCG Institutional Review Board (#1-1404234-1), and this research has been conducted according to principles with origin in Declaration of Helsinki.

MGPT on extracted DNA was performed using short-read massively parallel sequencing assays at Blueprint Genetics laboratory using Illumina NextSeq™ or NovaSeq™ sequencing systems. One of two panel tests were used: the Cystic Kidney Disease Panel (including up to 43 genes) or the Polycystic Kidney Disease Panel (including up to 13 genes). Test selection for each patient was at the clinician’s discretion based on their clinical judgement. Panel gene content changed over time as new gene discoveries were made and panels were updated (Table S1).

Both sequence variant and CNV analyses of massively parallel sequencing data were performed, targeting coding exons and 20 base pairs (bp) from the intron/exon boundaries. In addition, assays were customized with oligonucleotides targeting known disease-associated deep intronic and regulatory variants (Table S2). Sequence reads of each sample were mapped to the human reference genome (GRCh37/hg19). Sanger sequencing or digital or quantitative polymerase chain reaction methods were used to orthogonally confirm variants that did not meet rigorous internal sequencing quality score thresholds. The quality criteria included a variant call quality score, genomic location of the variant, sequence content, and visual analysis. These criteria were based on the outcome of an internal validation performed in the CLIA- and College of American Pathologists (CAP)-accredited Blueprint Genetics laboratory. Additionally, all variants reported as positive findings from DNA extracted from prenatal samples from ongoing pregnancies were orthogonally confirmed. The sequence variant analysis and CNV analysis pipelines were validated in the CLIA-certified, CAP- and ISO15189-accredited Blueprint Genetics laboratory. Variant classification and reporting were performed using a schema that aligns to the American College of Molecular Genetics and Genomics/Association for Molecular Pathology (ACMG/AMP) guidelines.^27^

For the purposes of this study, a positive result was defined as the identification of one pathogenic (P) or likely pathogenic (LP) variant consistent with the patient’s reported phenotype in genes associated with autosomal dominant or X-linked conditions or two P or LP variant(s) consistent with the patient’s reported phenotype in genes associated with autosomal recessive conditions. Cases with a single heterozygous pathogenic or likely pathogenic variant in a gene associated only with an autosomal recessive condition were not included among those with positive results. An inconclusive result is noted when variants were included on a patient’s report, but they did not meet the criteria to be considered a positive result. An inconclusive result is defined separately from a negative result, in which no variants were included on a patient’s report, for the purposes of this study. Variants of uncertain significance that were suspicious for being pathogenic (susVUS) were defined as having all of the following characteristics: (1) a strong and specific link between the gene and patient’s phenotype; (2) the variant is novel or extremely rare in the Genome Aggregation Database (gnomAD) control cohorts; (3) in silico predictions supporting pathogenicity or the amino acid position in question being highly conserved in mammals and evolutionary more distant species, suggesting that the position does not tolerate variation; and (4) demonstration of the variant as de novo or confirmation that the variants were in *trans* would likely result in reclassification to LP. Cohort demographics and positive rates were calculated using descriptive statistics and chi-square (χ^2^) analyses were used to assess significance.

## Results

A total of 1,235 consecutive reports from patients suspected to have CyKD and who underwent CyKD MGPT were reviewed (Table 1). Patient sex, as indicated by the clinician at the time of test order, was female (52.2%, n = 645), male (46.0%, n = 568), or unreported (1.8%, n = 22; 21 fetal and 1 child). The cohort included 875 (70.9%) adults, 320 (25.9%) children, and 40 (3.2%) fetuses.

**Table 1:** Cohort Population Demographics

Demographics	Number of Patient Reports	Proportion of Total Cohort (n = 1,235)
Sex as stated on test requisition		
Female	645	52.2%
Male	568	46.0%
Not reported	22	1.8%
Age range at time of testing		
Fetus	40	3.2%
Pediatric (0-18 years)	320	25.9%
Adult (19+ years)	875	70.9%
Panel		
Cystic Kidney Disease	420	34.0%
Polycystic Kidney Disease	815	66.0%

**Figure 1:**
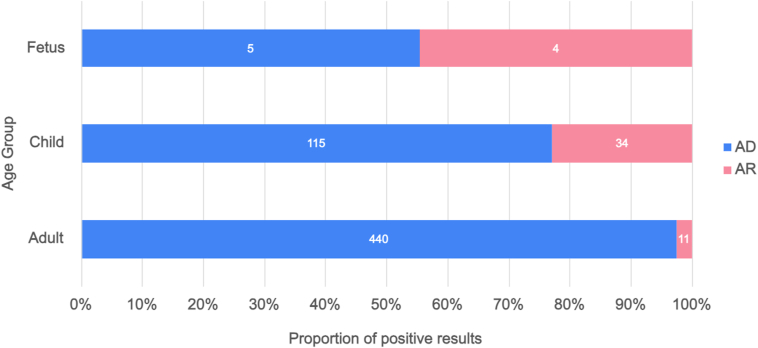
Proportion of positive results by mode of inheritance for each age group. Proportion of cases with positive results in genes associated with autosomal dominant conditions are represented in blue and the proportion of cases with positive results in genes associated with autosomal recessive conditions are pink. Abbreviations: AD, autosomal dominant; AR, autosomal recessive.

Positive results were identified in 49.4% (610/1235) of reports, with the identification of 468 unique variants classified as pathogenic or likely pathogenic (Tables S3 and S4) in 20 unique genes (Table 2). The most common genes in which a positive result was identified were *PKD1* (n = 400/610, 65.6%), *PKD2* (n = 93, 15.2%), *HNF1B* (n = 41, 6.7%), and *PKHD1* (n = 34, 5.6%). Sixteen different genes (*PRKCSH, PAX2, UMOD, SEC63, DNAJB11, GANAB, JAG1, NOTCH2, VHL, LRP5, NPHP1, NPHP3, INVS, NPHP4, WDR19,* and *OFD1*) each contributed to 1.0% or less of the total positive results, but cumulatively represented 6.9% (42/610) of positive results (Table 2). Positive results were identified in nine different genes exclusively offered on the Cystic Kidney Disease Panel (*INVS, NPHP1, NPHP3, NPHP4, OFD1, PAX2, UMOD, VHL,* and *WDR19*); these results represented 4.6% (28/610) of the total positive reports. Of Cystic Kidney Disease Panel reports, 39.3% (165/420) had positive results, whereas 54.6% (445/815) of Polycystic Kidney Disease Panel reports had a positive result. The yield of positive results was significantly higher among adults (51.7%, 452/875) and children (46.6%, 149/320) than for fetal cases (22.5%, 9/40); X^2^ (2, N = 1,235) = 14.4, *P* < 0.001. There was a significant relationship between the frequency of positive results associated with autosomal recessive (AR) conditions and age group; *X*^2^ (2, N = 610) = 84.6, *P* < 0.001 (Table 2). The fetal case group had the highest frequency of positive results related to AR conditions (44.4%, 4/9), whereas the pediatric case group and adult case group had frequencies of 24.2% (36/149) and 2.4% (11/452), respectively (Fig 1).

**Table 2:** Frequency and Proportion of Genes in Which Variants are Identified as Positive Results, Stratified by Modes of Inheritance of Associated Genetic Conditions

Gene	MOI	Number of patient reports	Proportion of patient reports with positive results by MOI (AD n = 560, AR = 49, XL = 1)	Proportion of patient reports with positive results (n = 610)
*PKD1*	AD	400	71.4%	65.6%
*PKD2*	AD	93	16.6%	15.2%
*HNF1B*	AD	41	7.3%	6.7%
*PRKCSH*	AD	6	1.1%	1%
*PAX2*	AD	6	1.1%	1%
*UMOD*	AD	5	0.9%	0.8%
*SEC63*	AD	3	0.5%	0.5%
*DNAJB11, GANAB, JAG1, NOTCH2, VHL, LRP5*	AD	6 (1 each gene)	1.1% (0.2% each gene)	1% (0.2% each gene)
*PKHD1*	AR	34	69.4%	5.6%
*NPHP1*	AR	5	10.2%	0.8%
*NPHP3*	AR	5	10.2%	0.8%
*INVS*	AR	3	6.1%	0.5%
*NPHP4, WRD19*	AR	2 (1 each gene)	4.1% (2% each gene)	0.3% (0.2% each gene)
*OFD1*	XL	1	100%	0.2%

Abbreviations: AD, autosomal dominant; AR, autosomal recessive; MOI, mode of inheritance; XL, X-linked.

**Figure 2:**
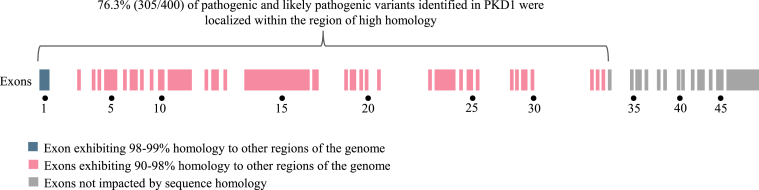
Representation of the *PKD1* gene and the homologous regions affecting exons 1 through 33, demonstrating the localization of identified pathogenic and likely pathogenic variants within the region of the gene affected by high homology to other regions of the genome

**Figure 3:**
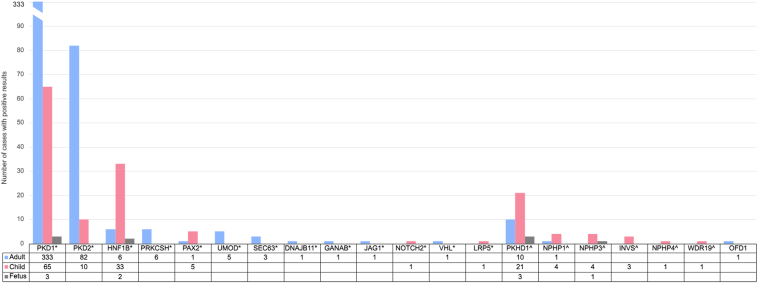
Number of cases with positive results in each gene, stratified by age group. Number of positive cases in the adult cohort is represented in blue, the child cohort in pink, and the fetal cohort in gray. The gene names are noted along the x-axis, annotated by ∗ for those associated with autosomal dominant conditions, ^ˆ^ for those associated with autosomal recessive conditions, and no annotation present for the gene associated with an X-linked condition. Below the gene names is a table with the number of cases with positive results in the gene for each age group.

P/LP variants in *PKD1* accounted for 65.6% of positive results (400/610) in the total cohort. Of these *PKD1*-related positive results, 95.5% (382/400) were sequence variants. Three *PKD1*-related positive results were a signature of several rare missense variants in the same region indicating a gene conversion event between *PKD1* and the pseudogene *PKD1P3.* These findings were confirmed via Sanger sequencing. Notably, 76.3% (305/400) of positive results in *PKD1* affected exons 1 through 33 of the NM_001009944.3 transcript (GrCH37/hg19 chr16:2138711-2147319), which are exons affected by sequence homology ranging from 98% in exon 1 to 90% to 98% in the remaining exons (Fig 2). *PKD1* variants contributed to a higher number of positive results in each age group than any other gene, representing 33.3% (3/9) of the positive results in the fetal group, 45.6% (68/149) of positive results in the pediatric group, and 72.8% (329/452) of positive results in the adult group (Fig 3).

CNVs were reported in 58/610 (9.5%) positive results: 56 were copy number losses (*HNF1B*, n = 28; *PKD1*, n = 17; *NPHP1*, n = 5; *PKD2*, n = 5; *PKHD1*, n = 1) and 2 were copy number gains (*PKD1*, n = 1; *HNF1B*, n = 1). The copy number losses (deletions) ranged in size from approximately 266 base pairs to 1.5 megabases. In total, 17.9% involved a single exon (10/56), 12.5% involved 2-4 exons (7/56), 8.9% involved 5 or more exons (5/56), and 60.7% affected at least one whole gene (34/56). The copy number gains (duplications) were both intragenic, spanning approximately 2.4 kilobases (2 exons of *HNF1B*) and 8.5 kilobases (8 exons of *PKD1*). Regarding *HNFIB* specifically, CNVs contributed to 70.7% (29/41) of positive results identified in this gene. Although the majority of CNVs in *HNF1B* affected the whole gene (n = 26), 2 patients were identified to have deletions affecting only 1 exon of the gene, and 1 patient was identified to have a duplication spanning approximately 2.4 kilobases that encompassed 2 exons of the gene. Conversely, only 2 of the 18 *PKD1* CNVs reported as positive results affected the entire gene with the remaining 16 being intragenic. Six patients had a deletion affecting a single exon in *PKD1*, ranging in size from approximately 266 to 912 base pairs and involving exons 1, 15, or 22 (NM_001009944.3). One patient had a gross deletion of at least 41.8 kilobases covering the first exon of *PKD1* and the 5' upstream region, including the transcription start site and the promoter; therefore, the deletion was predicted to impair gene expression or to result in loss of normal protein function.

Of the reports with inconclusive results, 57.0% (94/165) had a susVUS (Table S5). Segregation analysis of the variants identified in the patient in relevant family members could provide the additional evidence needed to reclassify the variant to likely pathogenic, allowing for a positive result. Of note, in 11 reports, a heterozygous susVUS in *PKHD1* associated with autosomal recessive disease was reported in addition to a heterozygous P/LP variant in the same gene. Parental testing to determine if these variants were on different parental alleles (in *trans*) would result in the susVUS being reclassified to LP, that is, a positive result.

## Discussion

These data contribute to the knowledge of genetic findings in large CyKD cohorts unselected for age and inheritance. This study demonstrates the utility of MGPT for the investigation of CyKD in an unselected referral population, with almost 1 of every 2 patients tested receiving a positive result. These findings are similar to the positive rates in cohorts receiving genetic testing for an indication of cystic kidney disease or to those with a broader indication of chronic kidney disease, ranging from 48% to 53%.^25^^,^^26^^,^^28^ These high positivity rates in our study, in addition to others published in the literature to date, may represent a bias in offering CyKD MGPT to patients with a compelling clinical presentation and/or family history and thus with a higher a priori risk of an underlying genetic cause. Additionally, in our cohort, 71.4% of positive results among genes associated with autosomal dominant disease were due to *PKD1*. This is similar to previous results that indicated *PKD1* underlies 78% of clinically diagnosed ADPKD.^29^

Positive findings in our cohort included variants in 20 different genes, illustrating the broad genetic heterogeneity underlying CyKDs. Variants in 16 unique genes individually contributed <1%, but collectively represented 7%, of the positive results in this cohort. These data collectively show that MGPT can simultaneously evaluate for common and rare molecular etiologies of CyKD.

Comprehensive MGPT also benefit individuals with atypical or complex disease presentations. For example, although it is recognized that ADPKD can present prenatally, it is most often expected to present in adulthood and therefore may not be considered a likely diagnosis when considering the differential for fetuses with CyKD on imaging.^30^, ^31^, ^32^, ^33^ However, more than half of positive findings in fetuses in this cohort (55.6%, n = 5/9) were due to variants in *PKD1* and *HNF1B*, genes responsible for autosomal dominant forms of CyKD. Conversely, although AR conditions are often associated with fetal and childhood diagnosis, 2.4% (11/451) of positive results in the adult cohort were due to variants in the genes associated with AR conditions, including *PKHD1* and *NPHP1* (Fig 3). The diversity in age of onset and potentially complex clinical presentations of CyKD reinforce the importance of including genes associated with both dominant and recessive conditions when selecting MGPT to shorten the diagnostic odyssey and increase the positive yield.

Given the morbidity and potential mortality related to extrarenal features of genetic conditions associated with CyKD in this cohort, the importance of understanding the genetic etiology of CyKD cannot be understated. For example, individuals with variants in *HNF1B,* associated with the variably expressed renal cysts and diabetes syndrome, may benefit from a referral to endocrinology for assessment for, or personalized management of, maturity-onset diabetes of the young (MODY). It is also important to recognize that the management implications for a biological female with cystic kidneys because of a variant in *OFD1*, which is associated with the variably expressed oral-facial-digital syndrome (OFDS) type 1, are dramatically different than for a biological female with cystic kidneys because of a variant in *PKD2*. OFDS warrants additional referrals to specialists for evaluation of neurological, skeletal, and oral extrarenal features for affected individuals. Further, OFDS is an X-linked condition that is typically gestationally lethal for affected males and thus has a significant impact on the reproductive care and counselling for affected women and their family members. These examples highlight that timely and accurate genetic testing for CyKD can influence extrarenal management for affected individuals and their family members, provide personalized guidance regarding appropriate specialty referrals, and have important implications for counseling about reproductive risks.

A wide variety of testing options are available to clinicians looking to integrate genetic analysis into their diagnostic workflow for patients with CyKD. Critical assessment of gene content, test methodology, and test capabilities before initiating testing for a patient is needed. MGPT designed for the indication of CyKD can vary in gene content and may not include genes associated with conditions considered within the ADPKD or ARPKD spectrums or disease mimics, such as *ALG5, ALG6, ALG8, ALG9, CLI2, COL4A3, COL4A4, COL4A5, DNAJB11, FLCN, GANAB, IFT140, LRP5, HNF1B, NEK8, OFD1, TSC1,* and *TSC2*.^13^^,^^15^^,^^20^^,^^34^^,^^35^ Although most clinical genetic testing laboratories include genes on their panels based on well-established gene-disease association, some laboratories may also include genes with limited or emerging evidence.^36^ Additionally, some tests may exclude well-characterized genes with clearly defined gene-disease associations in which analysis can be technically challenging, such as *PKD1*. As variants in *PKD1* were responsible for 65.5% of the positive results in this cohort and we see that 76.3% of these *PKD1* positive results were in the regions of the gene complicated by pseudogenes, exclusion of this gene or these technically challenging regions can have an important impact in identifying the molecular etiology for a patient’s CyKD. Lastly, the majority of genetic testing laboratories now include assessment for CNVs as part of their tests when using massively parallel sequencing methodology; however, laboratories may differ in their detection abilities, specifically for small CNVs (<4 exons or <1,000 base pairs in size).^37^^,^^38^ The frequency of CNVs in patients with CyKD has not been widely shared in available medical literature; however, we know that CNVs are a known mechanism of genetic forms of CyKD.^39^^,^^40^ Our finding that almost 1 in 10 positive results in this cohort included a CNV (9.5%, n = 59/620) offers novel insight into the value of optimized CNV detection using massively parallel sequencing workflows for maximizing the positive rate of genetic testing for CyKD. Further, it is essential that ordering providers choose a test that has robust abilities to assess for small CNVs to maximize the potential for positive results, as demonstrated by our report of 30.4% (17/56) of deletions identified as positive results consisting of four exons or less. Interestingly, the individuals identified to have the smallest deletions (200-400 base pairs) both had negative prior testing, including *PKD1* analysis, at other laboratories. These data reinforce the need for providers ordering genetic testing to not only be aware of panel content, but also sequencing capabilities for challenging genomic regions and copy number variants to ensure that the testing ordered is a comprehensive assessment of genes relevant to their patient.

Although most laboratories use ACMG/AMP guidelines for variant classification, which variants are reported back to the ordering clinician depends on laboratory policy.^27^ Variability in these reporting policies could result in a potentially clinically interesting variant being excluded from the report if it is currently classified as a variant of uncertain significance (VUS). Avoidance of VUS from clinicians and laboratories can have significant implications for patient care as follow-up family studies of susVUS results may provide evidence for variant reclassification to LP, allowing for a positive result. Frequency of VUS can vary widely by indication and ancestry. Although it has not been investigated specifically related to testing for CyKD, rates have been reported as high as 28% for a cohort evaluated for cardiac risk and as low as 9.2% among breast cancer patients.^41^^,^^42^ We identified suspicious VUS (susVUS) in 15.0% (94/625) of individuals without positive results in this cohort and importantly in 57.0% (94/165) of inconclusive reports. Although family member test results were not available at the time this manuscript was written, testing of family members demonstrating that the variant is de novo in the affected individual, segregation with disease among affected family members for autosomal dominant conditions, or biallelic occurrence for autosomal recessive conditions has the potential to reclassify these variants to likely pathogenic based on ACMG/AMP variant classification criteria and provide a positive result for these patients.^27^ Given the potential for additional positive results after variant reclassification, it is critically important for ordering clinicians to understand laboratory policy related to VUS reporting, how to recognize whether a reported VUS may have the potential to be reclassified, and the importance of a subsequent referral to genetics or follow up testing of family members for variants with the potential for reclassification.

Endeavors in assessing the appropriateness of various genetic tests, interpreting and disclosing positive results, discussing the implications of a VUS and organizing family member testing, and determining appropriate referrals may be daunting for some clinicians incorporating genetic testing into a busy clinic workflow. Enlisting the expertise of a trained medical genetics professional in the care of patients with CyKD is an option that can help reduce the load on nephrology teams. Genetic counselors are health care providers specifically trained in clinical genetics and offer expertise in assessing testing options and results disclosure in addition to an understanding of VUS and follow-up testing. Many labs offering clinical genetic testing also employ genetic counselors who are available to discuss results with the clinician and address questions. Alternatively, clinicians can refer patients to a genetic counselor or even integrate a genetic counselor into their own clinic. The involvement of genetic counselors in nephrology clinics has demonstrated success in identifying the most cost and time-effective genetic testing strategy, patient management, and provision of psychosocial support to families.^6^^,^^43^, ^44^, ^45^, ^46^

### Limitations

Several limitations exist as a result of the retrospective genetic testing report review study design. The availability of clinical information was limited to what was included on the test requisition form by the ordering clinician, which consequently precluded any correlation between genetic findings and clinical presentation. The ages reported for the participants of the study are ages at time of testing, not ages at time of first clinical presentation or ages at clinical diagnoses. The inability to identify if members of the same family were tested as part of this cohort and to rule out that the same individual was tested more than once could result in the inflation of frequencies of rare variants identified. Short-read, massively parallel sequencing methodology and exome-based target capture methodology may not detect, or may have limitations in the detection of, complex inversions, gene conversions, balanced translocations, and non-coding variants farther than ±20 base pairs from exon–intron boundary unless otherwise indicated (Table S2), low level mosaicism, stretches of mononucleotide repeats, indels larger than 50 bp, single exon deletions or duplications, and variants within pseudogene regions/duplicated segments. Finally, genetic analysis was limited to the genes included on the panel at the time of testing. Some genes that can mimic ADPKD, such as *COL4A3*, *COL4A4*, and *COL4A5* associated with Alport syndrome, were not standardly included as part of the analyses for individuals in these studies.^15^ As new evidence emerges supporting gene–disease associations underlying CyKD, such as the recently defined association of the *IFT140* gene with susceptibility to ADPKD, and panel content is updated, there is potential for additional positive results in those with previously inconclusive or negative results.^18^ The value of MGPT was not compared with other forms of genetic analysis, such as exome or genome sequencing, which interrogate more genes but have different limitations than MGPT.

## Conclusion

These results suggest that carefully curated and optimized next-generation sequencing-based multigene panels offer clinicians a valuable resource to determine the genetic etiology for individuals with cystic kidney disease. Given the findings of this retrospective review of genetic testing results, we suggest that multigene panel testing should be carefully evaluated, before test ordering, for factors including relevant gene content, capabilities for technically challenging genes like *PKD1,* sensitivity to detect copy number variants (especially those <4 exons in size), and the laboratory reporting policy for variants of uncertain significance. In addition, clinicians are encouraged to consider further work-up of suspicious variants of uncertain significance using family studies, if indicated, to maximize the opportunity for obtaining positive results using panel testing.
